# Known data on applied regenerative medicine in tendon healing

**DOI:** 10.6026/97320630017514

**Published:** 2021-04-30

**Authors:** Amit Lakhani, Ena Sharma, Amit Kapila, Kavin Khatri

**Affiliations:** 1 Dr Br Ambedkar State Institute of Medical Sciences, Mohali Punjab, India; 2 Maharishi Markandeshwar College of Dental Sciences and Hospital Mullana, Ambala, Haryana, India; 3 Consultant Fortis Hospital, Mohali, India; 4 All India Institute of Medical Sciences, Bathinda, Punjab, India

**Keywords:** Tendon, ligament, ACL, PRP, stem cells, scaffolds, gene therapy

## Abstract

Tendons and ligaments are important structures in the musculoskeletal system. Ligaments connect various bones and provide stability in complex movements of joints in the knee. Tendon is made of dense connective tissue and transmits the force of contraction from muscle to bone. They are injured due to direct trauma in sports or roadside accidents. Tendon healing after repair is often poor due to the formation of fibro vascular scar tissues with low mechanical property. Regenerative techniques such as PRP (platelet-rich plasma), stem cells, scaffolds, gene therapy, cell sheets, and scaffolds help augment repair and regenerate tissue in this context. Therefore, it is of interest to document known data (repair process, tissue regeneration, mechanical strength, and clinical outcome) on applied regenerative medicine in tendon healing.

## Background:

Tendon and ligament injuries are quite prevalent in the world. 33 million musculoskeletal impairments are recorded every year where about 50% are linked to tendons and ligaments in USA [1]. Walker *et al.* (2012) showed a loss of $27 million per annum due to sick leave for lateral epicondylitis (inflammation of an epicondyle) in the UK [2]. Conditions causing pain and reduced function of tendons are often referred as tendinopathy [3]. Effective strategy for the management of tendon injuries is limited [4]. Scleraxis (Scx) is a sclerotome marker and it is expressed in both tendon progenitor cells and mature tenocytes [5]. Fibroblast growth factor 8 (FGF8), secreted by the myotome, is partly responsible for inducing Scx expression through the Ets transcription factors Pea3 and Erm [6]. Growth and differentiation factors (GDF), members of the bone morphogenetic protein (BMP) family, are additional regulators of tendon development [7]. Tendons are enveloped by a layer of connective tissue known as endotenon that comprises of blood vessels, lymphatics, and nerves, to form larger structural units called fascicles, which are surrounded by another connective tissue layer called epitenon [8]. Type I Collagen is the fibril-forming collagen in tendons and co-polymerizes with collagen type V [9]. The type II transmembrane glycoproteins Tenomodulin (TNMD) is a marker for primed tenocytes and it is positively regulated by Scleraxis [10]. The natural healing process of tendons is extremely slow due to the hypo cellular and hypo vascular nature of tendon structure [11] with three stages: (a) inflammation, (b) repair and (c) remodelling [12] as shown in Figure 2. The inflammatory stage remains for 2 days followed by the repair and remodelling phase, which takes almost one year [13,14]. The role of various growth factors in the healing process of tendons [15]. There are various growth factors like insulin-like growth factor-I (IGF-I), TGF-β, bFGF, platelet-derived growth factor (PDGF), vascular endothelial growth factor (VEGF), BMP, and connective tissue growth factor (CTGF) which are particularly up-regulated following a tendon injury and are active at various stages of the healing process [16-19]. The current management plan for flexor tendon injuries including the post-operative plan to prevent re-rupture and hypertrophy of tendon is known [20]. However, a meta-analysis found rate of re-operation of 6%, re-rupture of 4%, and adhesion formation of 4% [21]. Achilles tendinopathy accounts for 40 to 50 % of sports injuries in young athletes [22]. Histo pathological studies have proved extensive degenerative changes in ruptured TA [23]. A failure rate of 5%-95% is observed for chronic tears in rotator cuff of shoulder joints [24]. The formation of fibro vascular scar tissue in place of a tough fibro collagenous band [25] due to the presence of anti-adhesive protein lubricin in synovial fluid [26] is seen in such cases. Therefore, it is of interest to document known data (repair process, tissue regeneration, mechanical strength, and clinical outcome) on applied regenerative medicine in tendon healing.

## Methodology

**Figure 1:**
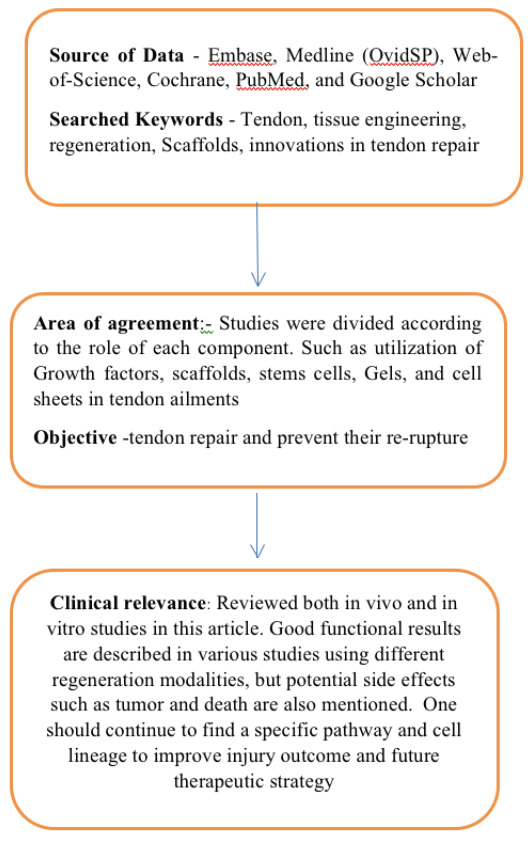
Methodology flowchart for data collection is shown

**Figure 2:**
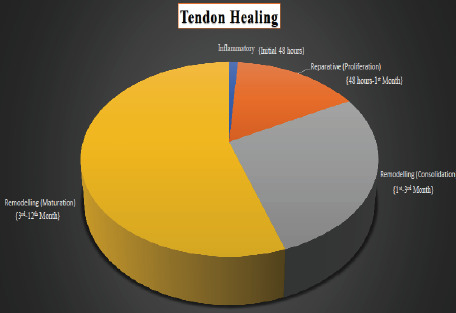
Stages in tendon healing is shown

The methodology for data collection is illustrated in Figure 1.

## Discussion:

### Methods for tendon repair and regeneration:

Current treatments for tendon repair and augmentation include biological grafts (e.g. auto grafts, allo grafts, and xeno grafts), prosthesis and tissue engineering. The biological grafts have several shortcomings as they induce donor site morbidity (auto graft) and tissue rejection (allograft). However, permanent prosthesis lack material durability causing mechanical malfunctions. Tendon tissue engineering (TTE) represents a most promising approach due to interdisciplinary engineering strategies. It aims to promote full tendon regeneration, rather than physically replacing tendons with partially functionalized foreign substitutes. TTE typically involves scaffolds, stem cells, gels, culture sheets, and gene therapy. TTE scaffolds can enhance tendogenesis by promoting cell proliferation, increasing matrix production, and organizing the matrix into functional tendon tissues. Moreover, tendogenesis can be facilitated through many strategies such as cellular hybridization, surface modification, growth factor cure, mechanical stimulation, and contact guidance.

### Growth factors:

Tendon injuries stimulates the increased expression of growth factors particularly in the early phases of healing. The growth factors that have shown a significant impact in tendon healing are bFGF, BMP-12, -13, -14, CTGF (connective tissue growth factor), IGF-1, PDGF, TGFβ, and VEGF. The role of these growth factors in tendon repair is extensively investigated [27-36]. The role of PRP (platelet-rich plasma derivative) has been analyzed in the field of orthopedics over a decade in human (Table 1 - see PDF). PRP is the plasma section of autologous blood containing a large concentration of platelets and growth factors such as platelet-derived growth factor (PDGF), transforming growth factor-beta (TGF-β), vascular endothelial growth factor (VEGF), epidermal growth factor (EGF), insulin-like growth factor-I (IGF-I), fibroblastic growth factor (FGF), and hepatocyte growth factor (HGF) [37]. Most of these factors promote neo-vascularization, tenocyte proliferation, and increase extracellular matrix production. PRP is prepared from autologous blood and it is inherently safe. PRP are present in physiological proportions with a natural balance of proliferative and inhibitory agents [38]. PRP preparation is made simple using advanced preparation devices. These technological advances have allowed PRP treatments to move from operating rooms to outpatient offices produced easily and safely in 15–30 minutes [39-45] (Table 2 - see PDF).

### Scaffolds:

The histological changes that are typical of the healed tendon are poor alterations in fiber structure, arrangement, vascularity, cellular morphology, and cellular proliferation. Scaffolds are placed into the defect zone to provide mechanical support and guide endogenous cells to improve matrix production and organization. Metcalf *et al.* described the use of porcine small intestinal submucosa (SIS) in 12 patients who underwent arthroscopic repair of massive chronic rotator cuff tears using Restore SIS as an augmentation device [46]. Postoperative magnetic resonance imaging (MRI) scans showed significant thickening of the cuff tendon with the incorporation of the SIS graft in 11 patients. However, worsening of symptoms in some patients due to SIS is also reported [47].

Acellular human dermal matrix [48], collagens repair patch [49], and polyfilamentous carbon composites [50] are various other alternative therapies giving promising results in human trials. Zimmer (USA) and De Tissue Science Laboratories, DePuy supply collagen repair patches for commercial purposes. The material is purified and cross-linked to collagenase degradation. Other therapies such as Type I collagen sponge [51] OFM (ovine fore stomach matrix [52]), fresh autograft fascia lata [53], PGA sheet [54] and polylactic acid patches [55] give good results in animal models. The findings of these studies are compelling and indicate the need for a long-term evaluation to verify the overall effectiveness of this augmentation method (Table 3 - see PDF).

###  Tendon gene therapy:

Gene therapy is the utilization of therapeutic nucleic acids into patient's cells to treat a disease condition. Tashjian *et al.* identified an SNP within the estrogen-related receptor beta (ESRRB) gene that appears to promote increased susceptibility to re-tears after a rotator cuff repair [56]. The molecular therapeutics and targeted gene therapies are the new frontiers in the treatment of rotator cuff disease [57]. Robertson et al found an increase in MMP1 and MMP9 gene expression in the patients with rerupture, compared to the group that displayed good healing [58]. The antibiotic doxycycline is an inhibitor of MMPs. Pasternak et al found that rat Achilles tendons repaired with doxycycline-coated sutures resulted in improved suture-holding capacity compared to a control group with uncoated sutures [59]. Current tissue engineering strategies using synthetic biomaterial scaffolds have yet to yield tendon substitutes. The appeal of these engineered scaffolds is that they can potentially be impregnated with growth factors or genes for targeted and timed-release at the site of implantation to improve healing. We reviewed 9 studies (Table 4 - see PDF) for the effect of various genes (rAAV-Gdf5, BMP-12, BMP-14 and PDGF) on tendon healing, strength, and movement [60-69]. This data is promising for further consideration.

### Stem cells:

Pluripotent stem cells carry great potential for cell therapy and tissue engineering. The use of embryonic stem cells (ESCs), adult mesenchymal stem cells (MSCs) tendon derived stem cells (TDSs), and Human skeletal muscle progenitor (SMP) cell to regenerate functional tendons and ligaments [70-79] (Table 5 - see PDF) is of interest. Various sources of MSCs have been investigated for their impacts on tendon repair. Embryonic stem cells (ESCs) have unlimited proliferation capacity and it can be induced into all types of somatic cells for tissue repair. However, there is a risk of teratoma formation. There are two promising cell types, namely bone marrow mesenchymal stem cells (BM-MSCs) and adipose-derived mesenchymal stem cells (AD-MSCs). They are well characterized and simple for in vitro proliferation. Interestingly, most of the preclinical animal studies concluded that MSC delivery can lead to increased cell proliferation, but these cells often differentiated towards osteoblasts or adipocytes within the tendon area, suggesting their inherent preference to commit to the original lineage of the tissue from which they were isolated [80]. The isolation of the native to the tendon-tenocytes, tendon stem/progenitor cells, or tendon-derived fibroblasts is relevant to the context [81]. MSCs have self-renewal and multilineage differentiation potential. BMSCs have shown immense collagen production after seeding on polylactide/glycolide (PLGA) suture material. Lee *et al.* [77] used Allogeneic adipose-derived mesenchymal stem cells in lateral epicondylosis and found tendon defect significantly reduced in 6 weeks. Ilic *et al.* studied mesenchymal stromal cells (MSCs) from the human placenta. They were injected directly into the site of tendon damage using ultrasound guidance in the treatment of chronic refractory tendinopathy and observed that there is significant improvement in tendon repair. Hernigou *et al.* [79] showed the role of crest bone marrow-derived mesenchymal stem cells (MSCs) in rotator cuff injury to prevent further damage.

### Gel and cell sheets:

Tendon repair and minor defects can be augmented with hydrogels with stem cells or direct cell sheets (Table 6 - see PDf). The tendon hydrogel promotes host cell infiltration, supporting its biocompatible properties and sustained the viability and proliferation of donor, adipose-derived stem cells (ASCs). The tendon hydrogel's thermo-property under physiologic temperature enhances its applicability in vivo. The gel polymerized and formed the shape of the defect at 37 degree Celsius. Hydrogel is a promising biomaterial for guided tissue regeneration. Degen *et al.* [82] showed rotator cuff repair augmentation with purified human MSCs with hydrogels in rat models. It was observed that there is improved early histologic appearance and biomechanical strength of the tendon at 2 weeks as described elsewhere [75-86]. Cell-cultured sheets derived from adipose stem cells, ACL, rotator cuff, and tendon stem cells were also used in this context despite increased cost [87-91].

### Amniotic membrane:

The epithelial and mesenchymal cells of amnion contain various regulatory mediators like Epidermal growth factor, Keratinocyte growth factor, a hepatocyte growth factor that results in the promotion of cellular proliferation, differentiation, epithelialization, inhibition of fibrosis, immune rejection, inflammation, and bacterial invasion (Table 7 - see PDF) [92]. The presence of platelet-derived growth factor (PDGF) and vascular endothelial-derived growth factor (VEGF) is suggestive of a pro-angiogenic role [93]. It is known that amniotic epithelial and mesenchymal cells lack HLA class A, B, DR, and co-stimulatory molecules CD-40, CD-80, and CD-86 making it non-immunogenic [94]. The effects of human amniotic fluid on peritendinous adhesion formation and tendon healing after flexor tendon surgery in rabbits are shown [95]. Amniotic membrane in flexor tendon repair has reduced adhesion [96]. Properties of the amniotic membrane for potential use in tissue engineering are available [97]. Flexor tendon repair using allograft amniotic membrane is also shown [98,99].

## Conclusion:

Known data (repair process, tissue regeneration, mechanical strength, and clinical outcome) on applied regenerative medicine in tendon healing is documented in this review. Information on the use of applied regenerative technologies such as the use of growth factors, scaffolds, gene therapy, stem cells, gel and cell sheets and amniotic membrane in tendon healing is gleaned from known literature to enrich our knowledge in this context. Caveats and limitations on known data including clinical trials, evidence based research information and FDA reveiws were found to be useful for further consideration [100-104].

## Ethical approval:

The Ethical committee of MMMCH at Kumarhatti Solan approved the review material.
